# Supplementary material for: The Evolution and Characterization of the RNA Interference Pathways in Lophotrochozoa
Source: Genome Biol Evol. 2024 May 7;16(5):evae098. doi: 10.1093/gbe/evae098 (PMC11114477; doi:10.1093/gbe/evae098)
Supplement: evae098_Supplementary_Data [file evae098_supplementary_data.zip › Supplementary_materials.zip!/selected_miRNAs/CrGi_selected_miRNAs/CrGi_NC_047561.1_12163.pdf]

Provisional ID	:	NC_047561.1_12163
Score total	:	10.9
Score for star read(s)	:	3.9
Score for read counts	:	4.2
Score for mfe	:	1.8
Score for randfold	:	1.6
Score for cons. seed	:	-0.6
Total read count	:	20
Mature read count	:	8
Loop read count	:	0
Star read count	:	12

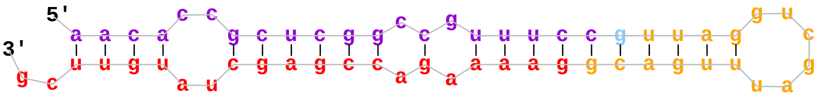

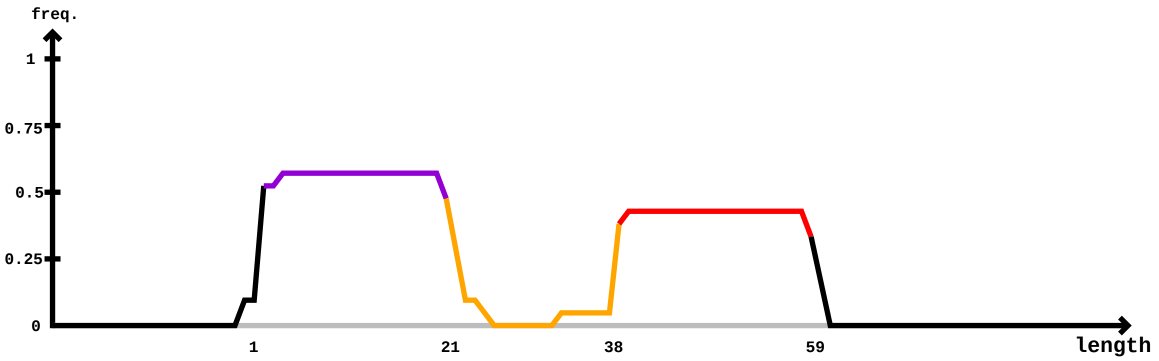

Star **Mature**

```
5'- caccuucugguuacaaucggaacaccgcucggccguuuccguuaggucgauuugacggaaaagaccgagcuauguucguauuguuuccggucagccaaaguaaaacaaaa  -3'   obs
    caccuucugguuacaaucggaacaccgcucggccguuuccguuaggucgauuugacggaaaagaccgagcuauguucguauuguuuccggucagccaaaguaaaacaaaa        exp
    ......((((..(((((..(((((..((((((.(.((((((((((......)))))))))).).))))))..)))))..)))))..))))....................   reads   mm   sample
    ..................ggaacaccgcucggccguuuc.......................................................................   1       0    Mt1
    .........................................................gaaaagaccgagcuauguuc.................................   1       0    Mt1
    .........................................................gaaaagaccgagcuauguucg................................   1       0    Mt1

    ....................aacaccgcucggccguuucc......................................................................   3       0    Mt2
    ....................aacaccgcucggccguuuccg.....................................................................   1       0    Mt2
    ....................aacaccgcucggccguuuccguu...................................................................   1       0    Mt2
    ......................caccgcucggccUuuuccguua..................................................................   1       1    Mt2
    .........................................................gaaaagaccgagcuauguucg................................   1       0    Mt2
    ..........................................................aaaagaccgagcuauguucg................................   1       0    Mt2

    ..................ggaacaccgcucggccguuuc.......................................................................   1       0    Ov2
    ....................aacaccgcucggccguuucc......................................................................   3       0    Ov2
    ...................................................uugacggaaaagaccgagcGauguuc.................................   1       1    Ov2
    .........................................................gaaaagaccgagcuauguucg................................   1       0    Ov2

    ....................aacaccgcucggccguuucc......................................................................   1       0    Ov1
    .........................................................gaaaagaccgagcuauguucg................................   3       0    Ov1
```
